# A putative glucose-1-phosphate thymidylyltransferase is required for virulence, membrane-associated mechanisms, and tolerance to external stresses in *Acidovorax citrulli*

**DOI:** 10.3389/fpls.2025.1556578

**Published:** 2025-05-21

**Authors:** Junhyeok Choi, Suhyun Lee, Dohyun Kim, Yoobin Han, Haerim Rhyu, Jisun H.J. Lee, Sang-Wook Han

**Affiliations:** Department of Plant Science and Technology, Chung-Ang University, Anseong, Republic of Korea

**Keywords:** *Acidovorax citrulli*, proteomic analysis, glucose-1-phosphate thymidylyltransferase, virulence, membrane integrity

## Abstract

Glucose-1-phosphate thymidylyltransferase (GptT) is crucial for bacterial cell wall/membrane functions. However, its roles in *Acidovorax citrulli* (*Ac*), the causative agent of bacterial fruit blotch (BFB) in watermelon, remain poorly understood. In this study, the roles of GptT in *Ac* (GptTAc) were elucidated through proteomic and phenotypic analyses using a mutant lacking GptTAc. The virulence of the mutant was remarkably reduced in the germinated-seed inoculation and leaf infiltration. However, its growth, as assessed by optical density (OD) in rich and minimal media, was comparable to that of the wild-type strain. A comparative proteomic analysis combined with clusters of orthologous group classification revealed that GptTAc was related to diverse mechanisms, including motility and the cell wall/membrane. The mutant showed increased lipopolysaccharide production but reduced exopolysaccharide production. Additionally, biofilm formation and auto-aggregation were enhanced, while twitching halo production was diminished. Notably, the mutant was highly susceptible to multiple stress conditions—including ethylenediaminetetraacetic acid, acetic acid, cupric chloride, sodium dodecyl sulfate, and pH stress—as indicated by significantly decreased OD values or colony forming units (CFUs) compared to the wild type. Finally, the mutant strain exhibited significantly higher sensitivity to lysozyme and antibiotics targeting the bacterial cell wall or membrane, as assessed by monitoring OD or CFUs, compared to the wild-type strain. Collectively, these findings suggest that GptTAc is involved in diverse cellular functions, particularly those related to cell wall/membrane integrity. This study provides novel insights into the role of GptTAc in the virulence of *Ac*, which may facilitate the identification of antivirulence agents targeting GptTAc by screening small-molecule and natural product libraries in order to control BFB.

## Introduction

*Acidovorax citrulli* (*Ac*) is a causative agent of bacterial fruit blotch (BFB), a devastating disease in cucurbits, including watermelon, that causes total crop loss in infected fields (Burdman and Walcott, 2012; Du et al., 2022). *Ac* strains are classified into two groups: group I is primarily associated with non-watermelon plants (mainly melon), while group II predominantly affects watermelon (Zivanovic and Walcott, 2017). *Ac* is primarily transmitted through seeds. Thus, infected seeds are the most significant source of BFB infection in fields where cucurbits are plated (Dutta et al., 2012). In watermelon, *Ac* infection initially manifests as wet lesions on the cotyledons and ultimately leads to the collapse and wilting of seedlings, culminating in cell death (Burdman et al., 2005). Infected watermelons exhibit dark, water-soaked lesions that can spread and cause secondary infections or ultimately result in fruit decay and collapse (Latin and Hopkins, 1995). Watermelon is one of the most extensively cultivated fruit crops in Korea, which ranked 24th among 107 countries in global production, with a total cultivation area of 11,762 ha in 2022 (Park et al., 2025). In Korea, BFB of watermelon was first reported in 1991 (Song et al., 1991). The disease was detected in most regions and caused significant crop losses during 2011–2012 (Noh et al., 2014). Despite the substantial threat posed by *Ac* to watermelon production, no commercial watermelon lines or varieties with resistance to *Ac* have been identified to date (Lee et al., 2024). Hence, the elucidation of mechanisms or factors related to virulence in *Ac* is required for exploring new approaches to control the disease.

Genes and mechanisms involved in virulence in *Ac* have been characterized using reverse and forward genetics. Genes related to protein secretion systems, including type II, III, and VI systems, have been reported as indispensable factors for the virulence of *Ac* (Tian et al., 2015; Zhang et al., 2018; Rosenberg et al., 2022; Kan et al., 2023). Biofilm formation and bacterial motility are also critical determinants of virulence (Bahar et al., 2009, 2010). Recently, a transcriptional regulator, *oxyR*, was reported to regulate both biofilm formation and motility, thereby contributing to virulence (Wang et al., 2022). In addition, the virulence-associated functions of genes involved in glycolysis, gluconeogenesis, primary metabolite synthesis, and nitrogen metabolism have also been documented (Liu et al., 2019; Lee et al., 2022b; Heo et al., 2023a, b; Liu et al., 2023b). Although several studies have investigated the virulence mechanisms of *Ac*, the functions of many genes/proteins associated with virulence remain to be elucidated for a comprehensive understanding of its pathogenicity. Therefore, further research is needed to elucidate the virulence factors and mechanisms in *Ac*.

Glucose-1-phosphate thymidylyltransferase (GptT) is a key enzyme involved in the biosynthesis of thymidine 5′-diphosphate (dTDP)-L-rhamnose, a crucial nucleotide sugar (Li et al., 2022). This biosynthetic pathway involves the coordinated action of four enzymes, including GptT. Although the genes encoding these enzymes are conserved across various species, their genomic organization can vary (Qu et al., 2007). The biosynthesis of dTDP-L-rhamnose is initiated at the nucleotide sugar level, commencing from glucose-1-phosphate (Ma et al., 1997). Through a series of enzymatic reactions, glucose-1-phosphate is converted into several dTDP-sugars, including dTDP-L-rhamnose, with the initial step catalyzed by GptT (Samuel and Reeves, 2003). L-rhamnose is widely found in the O-antigen of lipopolysaccharides (LPSs) in Gram-negative bacteria, and GptT has been well characterized for its role in LPS biosynthesis, particularly in animal pathogenic bacteria (Qu et al., 2007; Brown et al., 2018). However, its functional roles in plant pathogenic bacteria, including *Ac*, remain largely unexplored.

This study aimed to elucidate the function of a putative glucose-1-phosphate thymidylyltransferase in *Ac* (GptTAc). A mutant strain lacking functional GptTAc (*gptTAc:Tn5*) was generated, and its virulence was assessed in watermelon seedlings. To investigate the biological mechanisms underlying the function of GptTAc, a label-free comparative proteomic analysis was conducted using both the wild-type and mutant strains. Based on the proteomic data, diverse phenotypic assays, including bacterial motility, biofilm formation, LPS profiling, exopolysaccharide (EPS) production, and autoaggregation assays, were performed. Furthermore, the sensitivity of the mutant to ethylenediaminetetraacetic acid (EDTA), sodium dodecyl sulfate (SDS), acetic acid, cupric chloride, acidic and basic conditions, lysozyme, and antibiotics was evaluated. The results revealed that GptTAc is associated with diverse biological mechanisms, mainly cell wall/membrane function, contributing to virulence in *Ac*.

## Materials and methods

### Bacterial strains and growth conditions

*Ac* strain KACC17005 belonging to group II was used as the wild-type strain. The full genome of the strain was previously annotated (Park et al., 2017). *Escherichia coli* strains DH5α and EC100D were used for cloning and identifying the Tn5-insertional site, respectively. The *Ac* strains were grown in tryptic soy broth (TSB, 30 g/L) at 28°C, while the *E.coli* strains were grown in Luria–Bertani (LB) broth (1% tryptone, 0.5% yeast extract, and 1% NaCl) at 37°C. For antibiotic selection, all media used in the study were supplemented with the following final concentrations of antibiotics: 50 μg/mL rifampicin, 50 μg/mL kanamycin, 10 μg/mL gentamycin, and 100 μg/mL ampicillin.

### Identification of virulence-associated mutants and genetic complementation of *gptTAc: Tn5* strain

This experiment aimed to identify genes involved in the virulence of *Ac* and to validate their function through genetic complementation. The Tn5-insertional mutant library was screened using previously established protocols (Kim et al., 2020). To identify virulence-related genes, a germinated-seed inoculation assay was performed. Following the screening of approximately 200 mutants, two virulence-deficient mutants were identified, and their respective Tn5 insertion sites were determined according to the manufacturer’s protocol (Lucigen, Middleton, WI, USA). In one mutant, Tn5 was found to be inserted in a gene encoding a DNA-binding regulator, *hrpG* (Accession no. ATG95796), whose functions have been previously reported in *Acidovorax citrulli* (Zhang et al., 2018). In the other mutant, a gene encoding a putative GptT (Accession no. ATG96716) was disrupted by Tn5. Subsequently, this mutant was designated as *gptTAc:Tn5* To create a construct for complementation, the open reading frame of *gptTAc* was amplified using *gptTAc-*specific primers (forward: 5-gtcgacatgaccgcacgcaagggc-3; reverse: 5-tcaggttgctggccggtcaccaccaccaccaccactgaaagctt-3). The amplified DNA fragment was ligated into the pGem-T easy vector (Promega, Madison, WI, USA), generating pGem-*gptTAc*. After confirming the sequence using the Sanger method, the cloned DNA fragment was excised with *Sal*I and *Hind*III. The excised insert was then reintroduced into pBBR1-MCS5 (Kovach et al., 1995), which contains the *LacZ* promoter for controlling gene expression, generating pMCS5-GptTAc. The obtained construct was subsequently introduced into *gptTAc: Tn5*, creating *gptTAc:Tn5*(GptTAc). To avoid any side effects resulting from the vector, the empty vector was also introduced into *Ac* and *gptTAc: Tn5*, creating *Ac*(EV) and *gptTAc: Tn5*(EV), respectively. All plasmids and bacterial strains used in this study are listed in **Supplementary Table 1**.

### Assessment of virulence in watermelon using germinated seed inoculation and leaf infiltration methods

To investigate the involvement of GptTAc in virulence of *Ac*, germinated seed inoculation and leaf infiltration were conducted. *Citrullus lanatus* var. *vulgaris* line SBA (Partner Seed Co., Gimje, Republic of Korea) was used for virulence assays. Germinated seed inoculation was performed as per a previously established protocol (Heo et al., 2023a). The germinated seeds were soaked in 10 mM MgCl_2_ containing 10^6^ colony-forming units (CFU)/mL of *Ac* strains and incubated at 22°C for 1 h. Ten mM MgCl_2_, without any inoculum, was used as a negative control. The inoculated seeds were then moved to a controlled chamber under the following conditions: a temperature of 25 ± 1°C, 16-h light/8-h dark photoperiod, and relative humidity of 70%. Disease severity was scored for 7 days on a scale of 0 to 2. The experiment consisted of 10 biological replicates and was performed at least six times.


$$\begin{array}{c} Disease\;severity:\;\\ \frac{Normal(Plant_{n})\;\times \;0\;+\;Spot(Plant_{n})\;\times \;1\;+\;Wilt(Plant_{n})\;\times \;2}{Total(Plant_{n})} \end{array}$$

Leaf infiltration was performed as per a previously reported procedure (Heo et al., 2023b). In brief, the inoculum (approximately 10^5^ CFU/mL) was infiltrated into the second true leaf at the four-leaf stage of watermelon. Two leaf discs were excised using cork borers (0.4 cm in diameter), ground using sterilized water, serially diluted, and dotted onto TSA plates to measure the bacterial population (CFU). The population was measured at 2-day intervals for 8 days. Four independent experiments were conducted, with three biological replicates per strain.

### Comparative proteomic analysis to identify GptTAc-associated proteins in *Ac*

To investigate the impact of the *gptTAc* mutation on protein expression and to postulate biological mechanisms related to GptTAc, a label-free shotgun proteomic analysis combined with the cluster of orthologous groups (COG) classification was conducted as described previously (Lee et al., 2022a; Heo et al., 2023b). The three biological replicates of *Ac* and *gptTAc: Tn5* strains (six samples) were used for the comparative proteomic analysis. In brief, bacterial cultures grown in TSB were harvested at an OD_600 nm_ of 0.6, washed twice, and resuspended in 1 mL of suspension buffer containing 50 mM Tris–HCl (pH 7.8), 6 M guanidine HCl, and 10 mM dithiothreitol. The resuspended cells were lysed by sonication. After centrifugation, total soluble proteins were digested with trypsin. The concentration of the trypsin-digested peptides was measured using a BCA kit (Thermo Fisher Scientific, Rockford, IL, USA). The samples (1 μg) were then analyzed by liquid chromatography–tandem mass spectrometry (LC–MS/MS) using a split-free nano-LC system (EASY-nLC II, Thermo Fisher Scientific, Bremen, Germany) coupled with an LTQ Velos Pro instrument (Thermo Fisher Scientific). To obtain full mass spectra, the following parameters were applied. Six data-dependent MS/MS scans were performed per full scan. Dynamic exclusion was enabled with a repeat count of 1, a repeat duration of 0.5 minutes, and an exclusion duration of 3 minutes. Ion charge state selection was set to include 2+ and 3+ ions. For each full MS scan, the six most intense ions were selected for fragmentation. All analyses were conducted in centroid mode using the linear ion trap. The raw data generated from the LC–MS/MS analysis have been deposited in the ProteomeXchange Consortium via the PRIDE (Perez-Riverol et al., 2022) partner repository with the dataset identifier PXD059196.

The identification and quantification of proteins detected via the LC–MS/MS analysis were performed as described previously (Lee et al., 2022b). The SEQUEST search algorithm in Thermo Scientific Proteome Discoverer (ver. 1.3.0.399) was used to detect peptides and proteins based on the full genome sequence of *Ac* strain KACC17005 (accession no. CP023687). The target-decoy method was used to improve confidence (Elias and Gygi, 2007). The analyzed data were reimported into Scaffold 4 (Proteome Software, Portland, OR, USA) for comparing protein abundance. The peptide spectra match (PSM) values were used for comparison, as described previously (Choi et al., 2008). PSM values were calculated for each of the three biological replicates and normalized against the total PSM values. The average value of the replicates was used for determining the differential abundance of proteins (over twofold) between *Ac* and *gptTAc: Tn5*. Statistical analyses were performed using Student’s t-test to assess the significance of differences; p-values of <0.05 were considered statistically significant. Finally, the COG analysis (Tatusov et al., 2000) was carried out to classify differentially abundant proteins.

### Quantitative assessment of biofilm formation to evaluate the impact of the GptTAc

Biofilm formation was evaluated to assess the potential impact of GptTAc on surface attachment and community behavior, following a previously reported protocol (Heo et al., 2023b). Bacterial strains grown on a TSA plate were harvested and adjusted to an OD_600 nm_ of 0.3 (10^8^ CFU/mL). The samples were then serially diluted to 10^−2^ in TSB. The diluted samples (190 μL) were incubated in 96-well polyvinyl chloride (PVC) plates at 28°C. Biofilm formation was measured after 2 and 3 days. The bacterial cells attached to the well were stained with filtered 0.1% crystal violet and suspended in 95% ethanol (EtOH). The stained bacterial suspension was analyzed at 590 nm using a SpectraMax 190 microplate reader (Molecular Devices, Sunnyvale, CA, USA). A minimum of five independent experiments were conducted with 20 biological replicates.

### Assessment of twitching motility for halo formation analysis

To investigate whether the bacterial strains exhibit twitching motility, twitching halo formation was assessed following a previously established method (Heo et al., 2023b). Bacterial suspensions at an OD_600 nm_ of 0.3 (10^8^ CFU/mL) were diluted to 10^6^ CFU/mL. In total, 10 μL of the prepared sample was dropped onto TSA plates containing 0.5% agar and incubated at 28°C for 2 and 3 days. The colony and twitching halo sizes were assessed under a LEICA M205 C microscope (LEICA, Wetzlar, Germany). Five independent experiments were conducted with three biological replicates.

### Comparative growth analysis in nutrient-rich and minimal media

This experiment was conducted to compare the growth dynamics of bacterial strains under nutrient-rich and nutrient-limited conditions. Bacterial strains grown on TSA plates were harvested, suspended, adjusted to an OD_600 nm_ of 0.3 (10^8^ CFU/mL), and diluted to 10^6^ CFU/mL. To investigate the roles of GptTAc in bacterial multiplication, the growth of the bacterial cultures in TSB (nutrient-rich) was measured at 12-h intervals for 4 days using a spectrophotometer at OD_600 nm_. Three independent experiments were conducted with three biological replicates. To examine auxotrophic growth of the mutant, a M9 medium (47.7 mM Na_2_HPO_4_·7H_2_O, 22 mM KH_2_PO_4_, 8.6 mM NaCl, 18.7 mM NH_4_Cl, 2 mM MgSO_4_, 0.1 mM CaCl_2_, and 20 mL of 20% glucose in 1 L) was used. The bacterial suspension was adjusted to an OD_600 nm_ of 0.05 and incubated for 8 days. Growth was assessed at 1-day intervals over the specified period. The *Ac* strains were examined in three biological replicates. A total of three independent experiments were conducted.

### Quantitative evaluation of exopolysaccharide production

This experiment aimed to quantify the production of exopolysaccharides (EPS). To assess strain-specific differences in EPS production under defined growth conditions, an ethanol precipitation method was employed based on a previously described protocol with minor modifications (Ziadi et al., 2018). In brief, bacterial strains were incubated in TSB at an OD_600 nm_ of 0.1 and harvested at an OD_600 nm_ of 1 and 1.5. After collecting the chilled culture supernatant, two volumes of chilled EtOH were added to it. The mixture was thoroughly vortexed to form sufficient EPS–EtOH and then kept in a −20°C freezer overnight to facilitate precipitation. After performing centrifugation at 6000 ×g for 30 min at 4°C, the supernatant was completely removed and dried. The dried EPS was then weighed to determine its quantity. At least three independent experiments were conducted with three biological replicates.

### Evaluation of stress tolerance to determine the role of GptTAc in bacterial environmental adaptability

To determine whether GptTAc affects bacterial tolerance to various environmental stress conditions, stress tolerance assays were performed. Bacterial cells were grown in TSB supplemented with various stress-inducing agents: 0.5 or 1 mM EDTA, 15 mM acetic acid, 1 mM cupric chloride, 50 μg/mL vancomycin, 1 μg/mL polymyxin B, and TSB adjusted to different pH levels (5.0, 5.5, 6.0, 8.0, 9.0, and 10.0). Cultures were incubated for 96 hours at 28°C, and bacterial growth was monitored at 12-hour intervals by measuring optical density at OD_600 nm_ using a spectrophotometer. The experiments were conducted at least thrice with three biological replicates, to ensure reproducibility. For SDS, lysozyme, and penicillin treatments, the bacterial cells were suspended in TSB and adjusted to an OD_600 nm_ of 0.3. The cell cultures were treated with 0.01% SDS, 1 mg/mL lysozyme, and 100 μg/mL penicillin. Water was used as the negative control. The bacterial cultures were incubated for 1 (SDS and penicillin) or 2 (lysozyme) h, diluted serially, and dotted onto TSA plates to count viable cell numbers (CFU). Bacterial survival under the stress conditions was calculated based on the ratio of CFU under each stress condition to the CFU of the water control. At least three independent experiments were conducted with three biological replicates.

### Profiling and quantification of lipopolysaccharide to investigate the role of GptTAc

To investigate the effect of the GptTAc on LPS production, bacterial strains were cultured in 20 mL of TSB and harvested at an OD_600 nm_ of 1.0 by centrifugation. LPS from the samples was extracted using the LPS Extraction Kit (iNtRON Biotechnology, Seongnam, Republic of Korea), as per the manufacturer’s protocol. For LPS profiling, the extracted LPS were separated by tricine–SDS polyacrylamide gel electrophoresis and visualized using a silver staining kit (Pierce Silver Stain Kit, Thermo Fisher Scientific, Rockford, IL, USA). LPS quantification was performed using the phenol–sulfuric acid method, with slight modifications (Masuko et al., 2005). In brief, 50 μL of the extracted LPS was mixed with 150 μL of sulfuric acid and 5% phenol. After vortexing, the samples were placed at 90°C for 5 min. To measure the amount of LPS, the absorbance of the samples was measured at 490 nm using a spectrophotometer. Six independent experiments, each with three biological replicates, were conducted.

### Investigation of bacterial aggregation

To evaluate the impact of GptTAc on bacterial aggregation, a bacterial aggregation assay was conducted using a previously established protocol (Xiao et al., 2012). Bacterial cells grown on TSA were suspended in 10 mL of TSB at an OD_600 nm_ of 1.0 and incubated for 60 h without shaking. To monitor the bacterial population in the planktonic cells, 0.1 mL of the aqueous phase (approximately 2 cm below the surface) was collected. After adding 0.9 mL of water to the collected samples, the absorbance was measured at OD_600 nm_ using a spectrophotometer at 12-h intervals. Viable cell numbers were determined as CFU. At least three independent experiments were conducted with three biological replicates.

### Statistical analysis

Statistically significant differences were analyzed by one-way analysis of variance with Tukey’s HSD^ab^ test using SPSS 12.0K (Chicago, IL, USA). A p-value (below 0.05) represented a statistically significant difference.

## Results

### Identification of the *gptTAc* mutant and amino acid sequence comparison of GptTAc

On screening the Tn5-insertional mutant library, one mutant that did not exhibit disease symptoms was identified. In this mutant, a gene encoding GptT (accession no. ATG96716) was disrupted by a transposon. The deduced amino acid sequence of the identified gene was 85% and 87% similar to GptT sequences in *Salmonella enterica* (accession no. EAO9629959) and *E. coli* (accession no. MRF41702), respectively (**Supplementary Figure 1A**). Additionally, the predicted 3D structure of the gene generated using I-TASSER and PyMOL was highly similar to those of both *S. enterica* (accession no. EAO9629959) and *E. coli* (accession no. MRF41702) (**Supplementary Figure 1B**). Therefore, the gene and the mutant were named as *gptTAc* (glucose-1-phosphate thymidylyltransferase in *Ac*) and *gptTAc: Tn5*, respectively.

### GptTAc is essential for virulence

**Figure 1:**
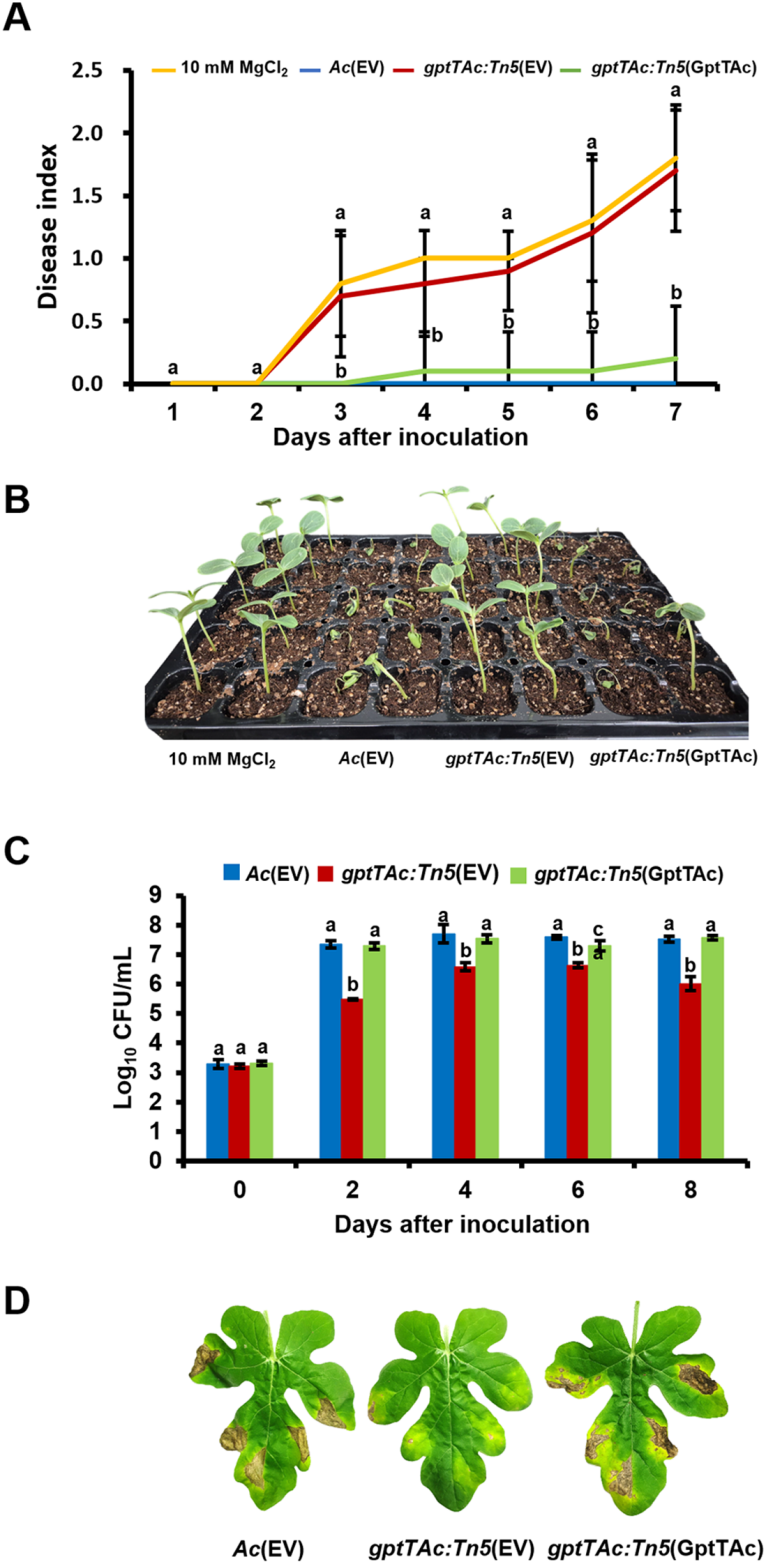
Virulence test of *Ac*(EV), *gptTAc: Tn5*(EV), and *gptTAc: Tn5(*GptTAc). **(A)** The disease index was calculated via germinated seed inoculation for 7 days. Ten mM of MgCl_2_ was used as a negative control. The following formula was used: [No disease (number of plants) × 0 + Spot (number of plants) × 1 + Wilt (number of plants) × 2]/Total (number of plants). Alphabets above error bars indicate statistical significance by ANOVA (p < 0.05) with Tukey’s HSD^ab^ test and error bars represent standard deviation from ten biological replicates. **(B)** A photograph from germinated seed inoculation was taken 7 days after inoculation. **(C)** Viable cell numbers were determined by counting colonies in the infected leaves for 8 days in the leaf infiltration assay. **(D)** Photographs depicting the condition of the leaves were taken 8 days after infiltration. Alphabets above error bars indicate statistical significance determined by ANOVA (p < 0.05) with Tukey’s HSD^ab^ test, and error bars represent standard errors of means. At least four independent experiments were conducted, displaying similar patterns.

Germinated-seed inoculation, and leaf infiltration on watermelon were performed to assess the role of GptTAc in virulence. A complemented strain, *gptTAc: Tn5*(GptTAc), in which the mutant carries *gptTAc* driven by the *lac* promoter on pBBR1-MCS5, was generated and used for the virulence assays. To eliminate any side effects resulting from the vector, *Ac*(EV) and *gptTAc: Tn5*(EV), which carry an empty vector in the mutant and wild-type strains, respectively, were also used for the assays. In the seed inoculation assay, the plants infected with *Ac*(EV) exhibited typical disease symptoms. All plants inoculated with *Ac*(EV) died at 7 DAI, and the disease index reached 2.0 (**Figures 1A, B**). However, the virulence of *gptTAc: Tn5*(EV) was abolished and the disease index of the plants infected with the mutant remained 0.2 at 7 DAI. The disease index in the negative control was 0.0 at 7 DAI. However, there was no statistical difference between the mutant and the negative control. The complemented strain *gptTAc: Tn5*(GptTAc) exhibited similar patterns to the wild-type strain, indicating that Tn5 caused no positional effects or side effects in the mutant. The results of the leaf infiltration assay exhibited similar patterns to those of the germinated seed inoculation assay (**Figures 1C, D**). The colony forming units (CFUs) in the *gptTAc: Tn5*(EV) group were dramatically lower than those in both *Ac*(EV) and *gptTAc: Tn5*(GptTAc) groups during the observation period. The leaves infiltrated with the wild-type and complemented strains exhibited tissue necrosis, appearing as dark brown patches, while those infected with the mutant exhibited very limited symptoms (**Figure 1D**).

### GptTAc is not associated with bacterial growth in media

The growth and virulence of the mutant were clearly reduced in watermelon in the two pathogenicity assays. To determine whether these reductions were attributed to the involvement of GptTAc in the growth of the mutant, the growth patterns of three bacterial strains, *Ac*(EV), *gptTAc: Tn5*(EV), and *gptTAc: Tn5*(GptTAc), were evaluated. In a nutrient-rich medium (TSB), all three strains displayed similar growth patterns, indicating that GptTAc is not required for the multiplication of *Ac* (**Supplementary Figure 2A**). Moreover, no significant differences were noted in the growth patterns of the three strains in a minimal nutrient medium (M9) (**Supplementary Figure 2B**), indicating that *gptTAc: Tn5*(EV) is not an auxotroph and GptTAc is not involved in the biosynthesis of primary metabolites.

### Label-free shotgun comparative proteomic analysis

**Figure 2:**
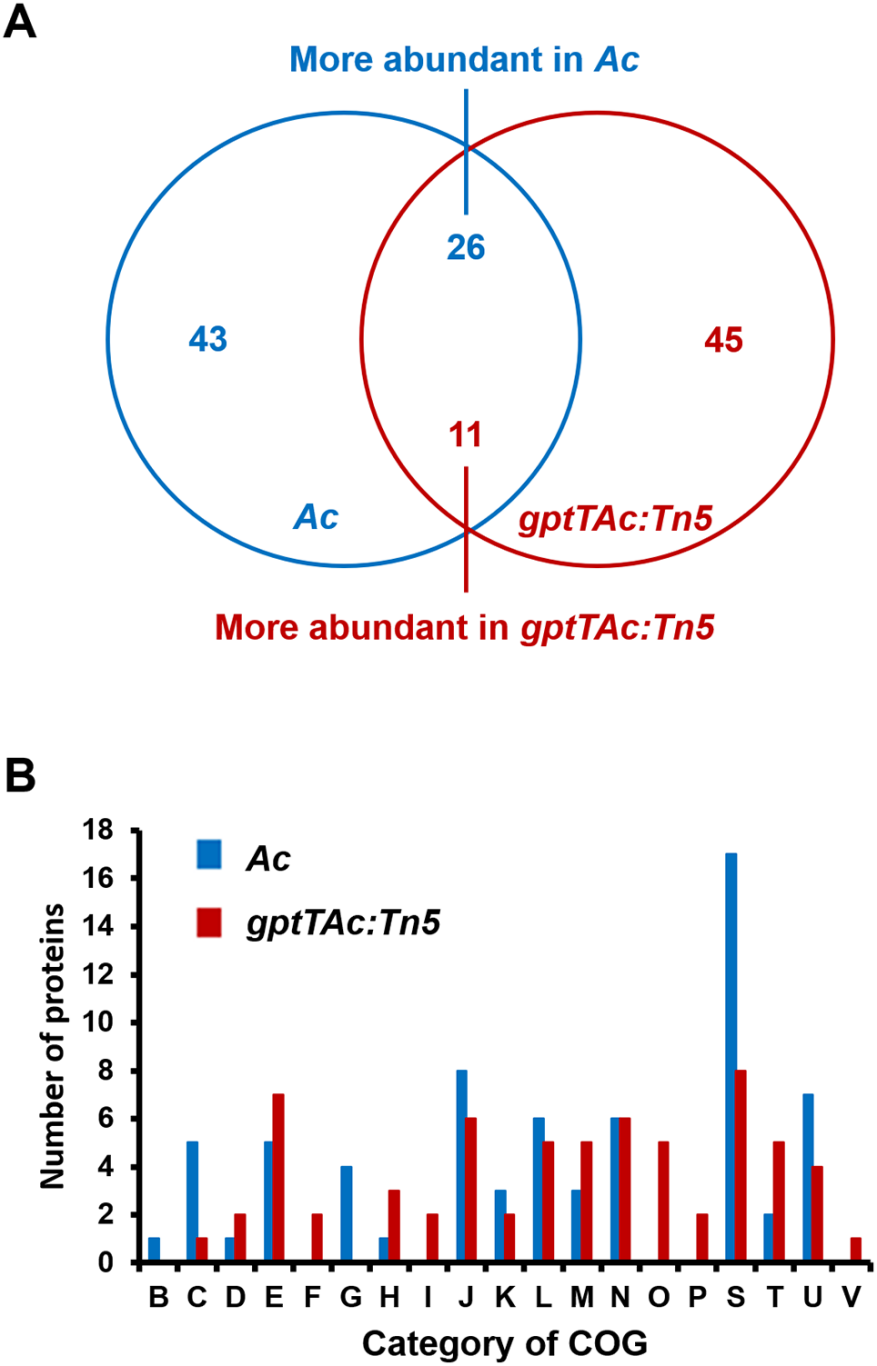
Comparative proteomic analysis between *Ac* and *gptTAc: Tn5*. **(A)** A Venn diagram showing numbers of differentially abundant proteins (over twofold change). In total, 43 and 46 proteins were exclusively detected in *Ac* and *gptTAc: Tn5*, respectively. Moreover, 26 and 11 proteins were more abundant in *Ac* and *gptTAc: Tn5*, respectively. **(B)** Categorization of differentially abundant proteins using Clusters of Orthologous Groups (COG) analysis. Group B, chromatin structure and dynamics; C, energy production and conversion; D, cell cycle control and mitosis; E, amino acid metabolism and transport; F, nucleotide metabolism and transport; G, carbohydrate metabolism and transport; H, coenzyme metabolism; I, lipid metabolism; J, translation; K, transcription; L, replication and repair; M, cell wall/membrane/envelope biogenesis; N, cell motility; O, post-translational modification, protein turnover, and chaperones; P, inorganic ion transport and metabolism; S, function unknown; T, signal transduction; U, intracellular trafficking and secretion; and V, defense mechanisms.

In addition to assessing the requirement of GptTAc for virulence in *Ac*, the possible mechanisms related to GptTAc were assessed in *Ac* and *gptTAc: Tn5* strains via a label-free shotgun comparative proteomic analysis. In the LC–MS/MS analysis, 687 and 691 proteins were commonly found in the three biological replicates of *Ac* and *gptTAc: Tn5*, respectively (**Supplementary Table 2**). Among the proteins shared between *Ac* and *gptTAc: Tn5*, proteins showing a statistically significant difference in abundance, with changes exceeding two-fold, were selected. On comparing protein abundance, 43 and 45 proteins were specifically identified in *Ac* and *gptTAc: Tn5*, respectively. Moreover, 26 and 11 proteins were more abundant (at least two-fold higher) in *Ac* and *gptTAc: Tn5*, respectively (**Figure 2A**). Notably, GptTAc was detected in the wild-type strain but not in the mutant, indicating that the mutant does not produce GptTAc. The comparative proteomic analysis was thus well-established. Next, these identified proteins were analyzed by performing the COG analysis to postulate the mechanisms related to GptTAc (**Figure 2B**; **Supplementary Tables 3**, **4**). Within the COG classification, groups B (chromatin structure and dynamics) and G (carbohydrate metabolism) were exclusively observed in *Ac*, while groups F (nucleotide metabolism and transport), I (lipid metabolism), O (post-translational modification, protein turnover, and chaperones), P (inorganic ion transport and metabolism), and V (signal transduction) were exclusively identified in *gptTAc: Tn5* (**Figure 2B**). The categories with the highest number of proteins were J (translation), E (amino acid metabolism and transport), N (cell motility), L (replication and repair), U (intracellular trafficking and secretion), and M (cell wall/membrane/envelope biogenesis) (14, 12, 12, 11, 11, and eight proteins, respectively). Notably, groups M and N accounted for 14.81% of the total COG classification (**Figure 2B**). The proteomic analysis revealed notable differences between group M, which appears to have the greatest impact on biofilm formation and extracellular stress response, and group N, which plays a crucial role in pathogenicity and cell motility.

### *gptTAc: Tn5* enhances LPS production and biofilm formation but reduces EPS production

**Figure 3:**
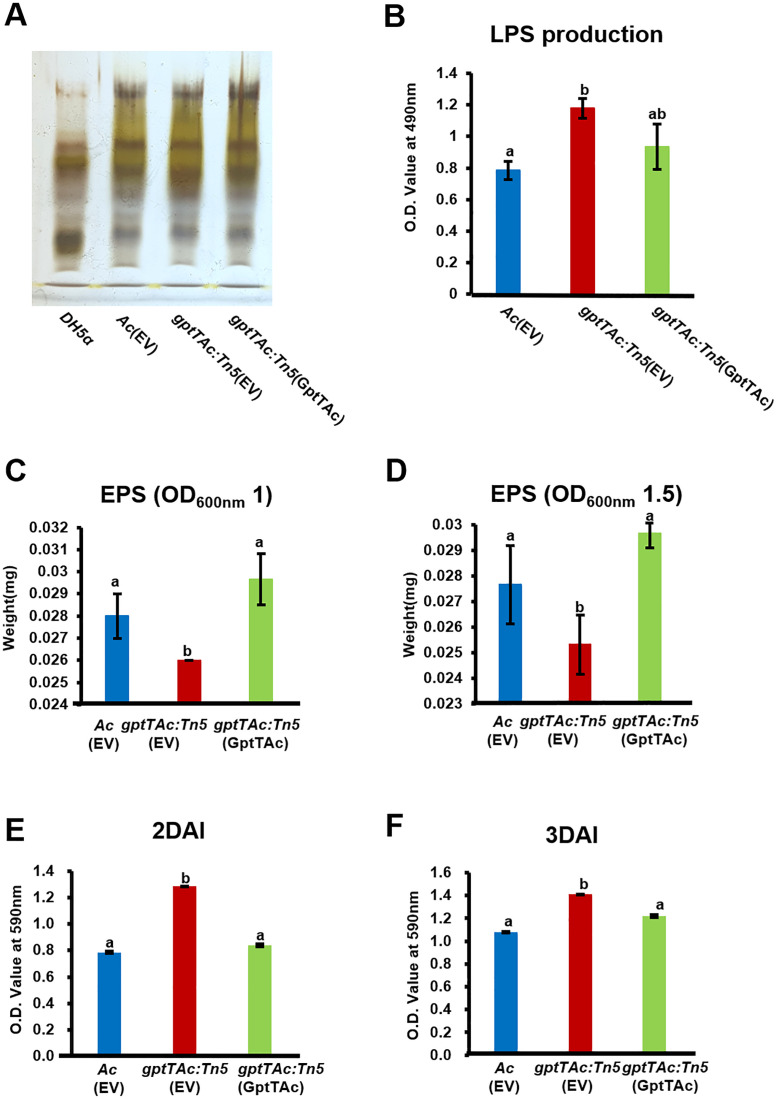
Lipopolysaccharide, extrapolysaccharide, and biofilm formation by *Ac*(EV), *gptTAc: Tn5*(EV), and *gptTAc: Tn5(*GptTAc). **(A)** LPS profiling on tricine–SDS PAGE: Line 1, DH5a; Line 2, *Ac*(EV); Line 3, *gptTAc: Tn5*(EV); and Line 4, *gptTAc: Tn5*(GptTAc). **(B)** Quantification of LPS sugar content using the phenol–sulfuric acid method. Quantification of EPS from *Ac* strains collected at an OD_600 nm_ of **(C)** 1.0 and **(D)** 1.5. Measurement of biofilm quantity at **(E)** 2 and **(F)** 3 days after incubation (DAI) in a 96-well polyvinyl chloride plate. Alphabets above error bars indicate statistical significance determined by ANOVA (p < 0.05) with Tukey’s HSD^ab^ test, and error bars represent standard errors of means. At least three independent experiments were conducted, and all experiments showed similar patterns.

Both LPS and EPS are closely associated with bacterial membrane functions, and several proteins classified under group M (cell wall/membrane/envelope biogenesis) were identified through comparative proteomic analysis. Therefore, the profiles and amounts of LPSs produced by *Ac*(EV), *gptTAc: Tn5*(EV), and *gptTAc: Tn5*(GptTAc) were assessed. The profiles of LPSs produced by the three strains exhibited similar patterns (**Figure 3A**). However, the LPS amount was higher in the mutant than in the wild-type and complemented strains (**Figure 3B**). EPS production was also evaluated. The EPS content in *gptTAc: Tn5*(EV) was lower than that in the wild-type strain (7.2% lower at OD 1.0 and 10.1% lower at OD 1.5) (**Figures 3C, D**). The EPS level in the complemented strain *gptTAc: Tn5*(GptTAc) was restored to that of the wild-type strain. This intricate phenomenon tightly interweaves with LPS and extracellular polymeric substances. As *gptTAc: Tn5*(EV) exhibited abnormal LPS production, biofilm formation in the three strains was also assessed in a 96-well PVC plate. The mutant exhibited significantly higher biofilm formation than the wild-type strain at both 2 and 3 days after incubation (DAI) (**Figures 3E, F**). The biofilm formation ability of *gptTAc: Tn5*(GptTAc) was similar to that of *Ac*(EV).

### *gptTAc: Tn5* reduces twitching motility and enhances cell aggregation

**Figure 4:**
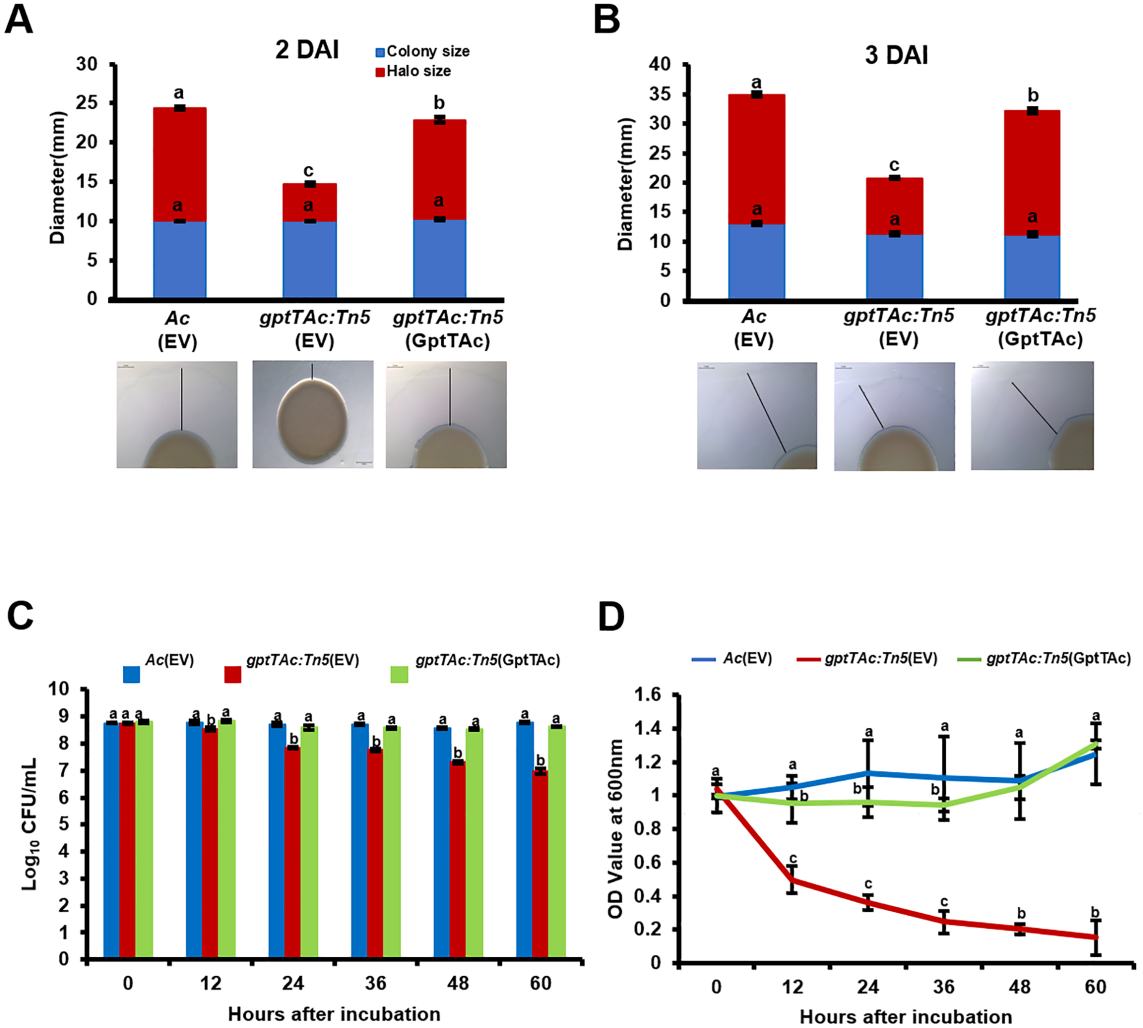
Twitching halo production and autoaggregation in *Ac*(EV), *gptTAc: Tn5*(EV), and *gptTAc: Tn5(*GptTAc). **(A)** Diameters of colonies and halos at **(A)** 2 and **(B)** 3 days after incubation. The scale bar represents 2 mm. **(C)** Viable cell numbers and **(D)** OD values of *Ac* strains at a depth of 1 cm below the surface assessed for 60 h on incubation in 14 mL test tubes containing TSB. Alphabets above error bars indicate statistical significance determined by ANOVA (p < 0.05) with Tukey’s HSD^ab^ test, and error bars represent standard errors of means. At least four independent experiments were conducted, showing similar results.

In the comparative proteomic analysis, several proteins classified in group N (cell motility) were also detected. Therefore, pilus-dependent motility, specifically twitching motility, was tested on a semisolid TSA plate containing 0.5% agar. No significant difference was noted in the diameters of colonies produced by *Ac*(EV), *gptTAc: Tn5*(EV), and *gptTAc: Tn5*(GptTAc) at 2 and 3 DAI, indicating that the growth of the three strains did not differ (**Figures 4A, B**). Interestingly, the size of the twitching halo in the mutant was dramatically lower than those in the wild-type and complemented strains during the observation period.

Therefore, the dynamics of bacterial aggregation were assessed by performing a sedimentation assay of bacterial cells in TSB without shaking. In this assay, the CFU and OD values of freely floating cells in the aqueous phase were measured. The viable cell numbers of *Ac*(EV) and *gptTAc: Tn5*(GptTAc) did not change during the entire observation period (**Figure 4C**). However, in congruence with sedimentation, the population of *gptTAc: Tn5*(EV) in the aqueous phase exhibited a progressive decrease. The OD values of the mutant in the aquatic phase were drastically reduced during the observation period, while those of the wild-type and complemented strains were not (**Figure 4D**; **Supplementary Figure 3**).

### *gptTAc: Tn5*(EV) is more susceptible to EDTA and SDS

**Figure 5:**
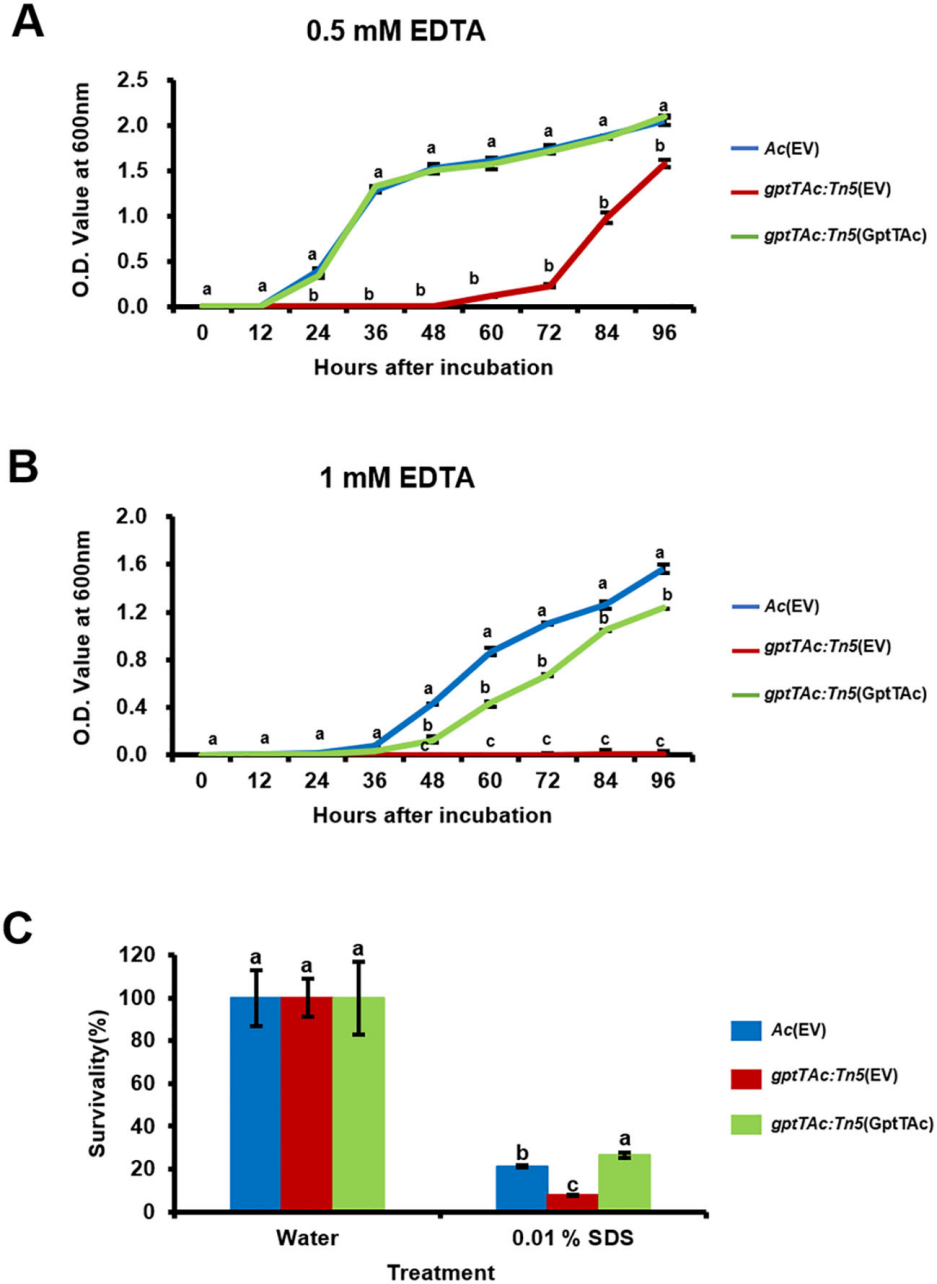
Tolerance assays to SDS and EDTA in *Ac* strains. Growth curves of *Ac*(EV), *gptTAc: Tn5*(EV), and *gptTAc: Tn5(*GptTAc) were established in TSB in the presence of **(A)** 0.5 mM and **(B)** 1 mM EDTA using a spectrophotometer for 96 h **(C)** Survivability of *Ac* strains exposed to 0.01% SDS for 1 h Survivability was compared to the water control using a colony counting method. Error bars represent standard errors of means. Alphabets above error bars indicate statistical significance determined by ANOVA (p < 0.05) with Tukey’s HSD^ab^ test. At least three independent experiments were conducted, and all results exhibited similar patterns.

The comparative proteomic analysis implied that GptTAc is associated with the functions of the bacterial cell/membrane. Therefore, the growth patterns of *Ac* strains were examined in TSB containing EDTA. In the presence of 0.5 mM EDTA, *gptTAc: Tn5*(EV) displayed significantly greater sensitivity than the wild-type and complemented strains (**Figure 5A**). Notably, in the presence of 1 mM EDTA, the mutant did not exhibit any noticeable growth (**Figure 5B**), suggesting a profound impact of EDTA on viability. In addition to EDTA, the tolerance to SDS was investigated because SDS acts on the hydrophobic tails of lipid bilayers in cell membranes and induces instability in bacterial membranes (Sudbrack et al., 2011). In the presence of 0.01% SDS, the survivability of the mutant (8.06%) was significantly lower than that of the wild-type strain (21.2%); however, the survivability was restored in the complemented strain (26.6%) (**Figure 5C**).

### *gptTAc: Tn5*(EV) reduces tolerance to lysozyme, penicillin, vancomycin, and polymyxin B

**Figure 6:**
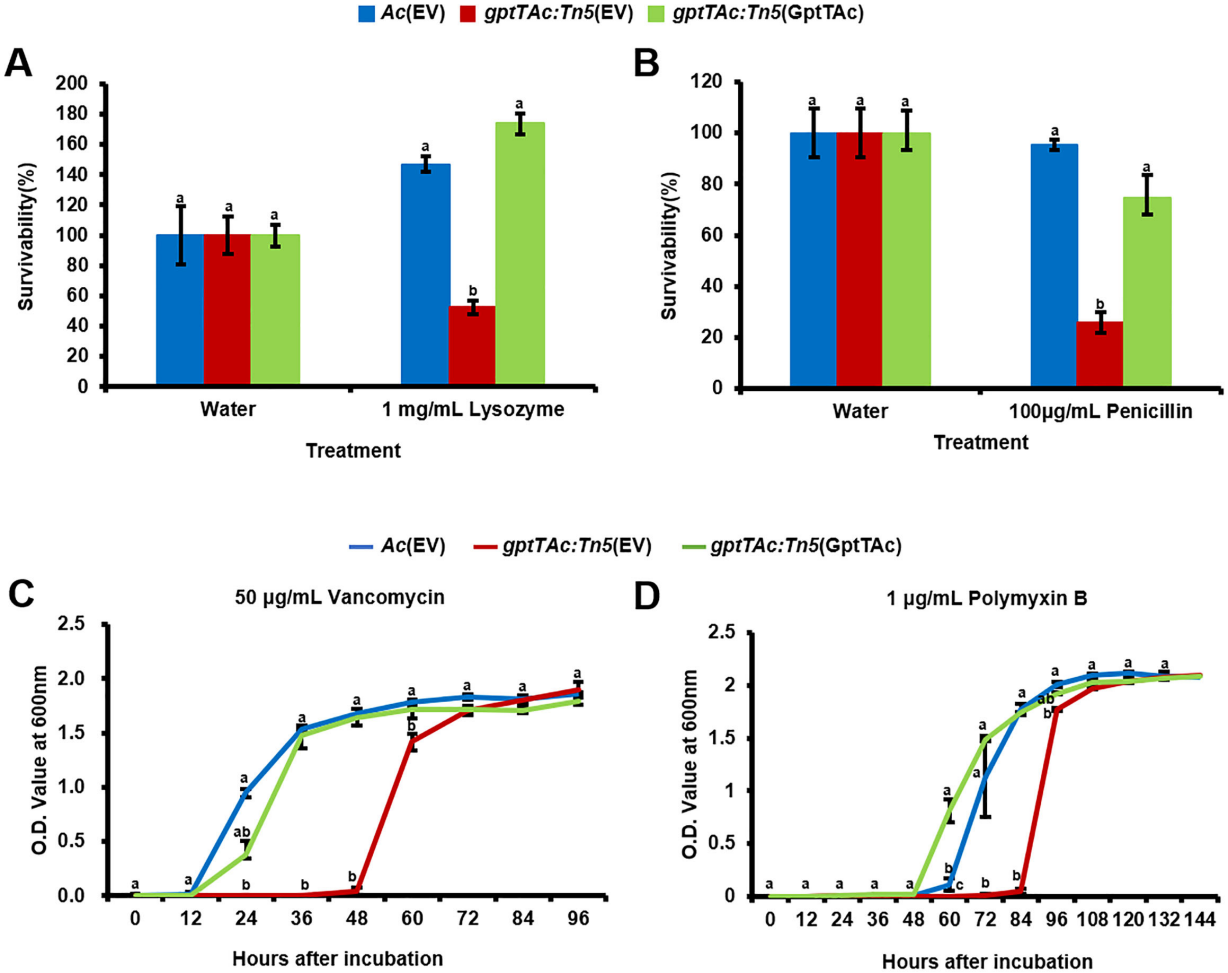
Sensitivity assays to lysozyme, penicillin, vancomycin, and polymyxin B in *Ac* strains. Survivability of *Ac*(EV), *gptTAc: Tn5*(EV), and *gptTAc: Tn5(*GptTAc) exposed to **(A)** 1 mg/mL lysozyme for 2 h and **(B)** 100 μg/mL penicillin for 1 h was determined using a colony counting method and calculated by comparison to the water control. Bacterial growth in TSB in the presence of **(C)** 50 μg/mL vancomycin for 96 h and **(D)** 1 μg/mL polymyxin B for 144 h was measured using a spectrophotometer. Error bars represent standard errors of means. Alphabets above error bars indicate statistical significance determined by ANOVA (p < 0.05) with Tukey’s HSD^ab^ test. At least three independent experiments were conducted, exhibiting similar patterns.

In addition to the comparative proteomic analysis, phenotypic observations of sensitivity to EDTA and SDS demonstrated that GptTAc is associated with membrane integrity. Therefore, the tolerance to lysozyme and some antibiotics targeting bacterial membranes was assessed in *Ac*(EV), *gptTAc: Tn5*(EV), and *gptTAc: Tn5*(GptTAc). Lysozyme is an enzyme that acts on the glycosidic bonds between N-acetylglucosamine (NAG) and N-acetylmuramic acid (NAM) in peptidoglycans, disrupting the formation of the peptidoglycan layer (Wang et al., 2012). In the presence of 1 mg/mL lysozyme, the tolerance of *gptTAc: Tn5*(EV) was significantly lower than that of the wild-type strain. However, the tolerance of *gptTAc: Tn5*(GptTAc) was restored to the level noted in the wild-type strain (**Figure 6A**). Next, the sensitivity to penicillin, which is β-lactam antibiotics that bind to penicillin-binding proteins and interfere with peptidoglycan synthesis, was assessed. *gptTAc: Tn5*(EV) exhibited enhanced susceptibility to penicillin (**Figure 6B**). The complemented strain and wild-type strain exhibited a similar tolerance to penicillin. Subsequently, the sensitivity to vancomycin, which inhibits cell wall synthesis in gram-positive bacteria but not in gram-negative bacteria, was assessed Intriguingly, *gptTAc: Tn5*(EV) exhibited growth retardation in the presence of vancomycin (**Figure 6C**). In the wild-type and complemented strains, the lag phase lasted for approximately 12 h; however, the mutant exhibited an extended lag phase (approximately 45 h). Similar results were observed in the case of polymyxin B that directly disrupts bacterial outer cell membranes (**Figure 6D**). In the presence of polymyxin B (1 μg/mL), growth retardation was noted in *gptTAc: Tn5*(EV). The exponential phase in the mutant was significantly delayed compared with that in the wild-type and complemented strains.

### *gptTAc: Tn5*(EV) exhibits altered growth under acetic acid, cupric chloride, and acidic and basic stress conditions

**Figure 7:**
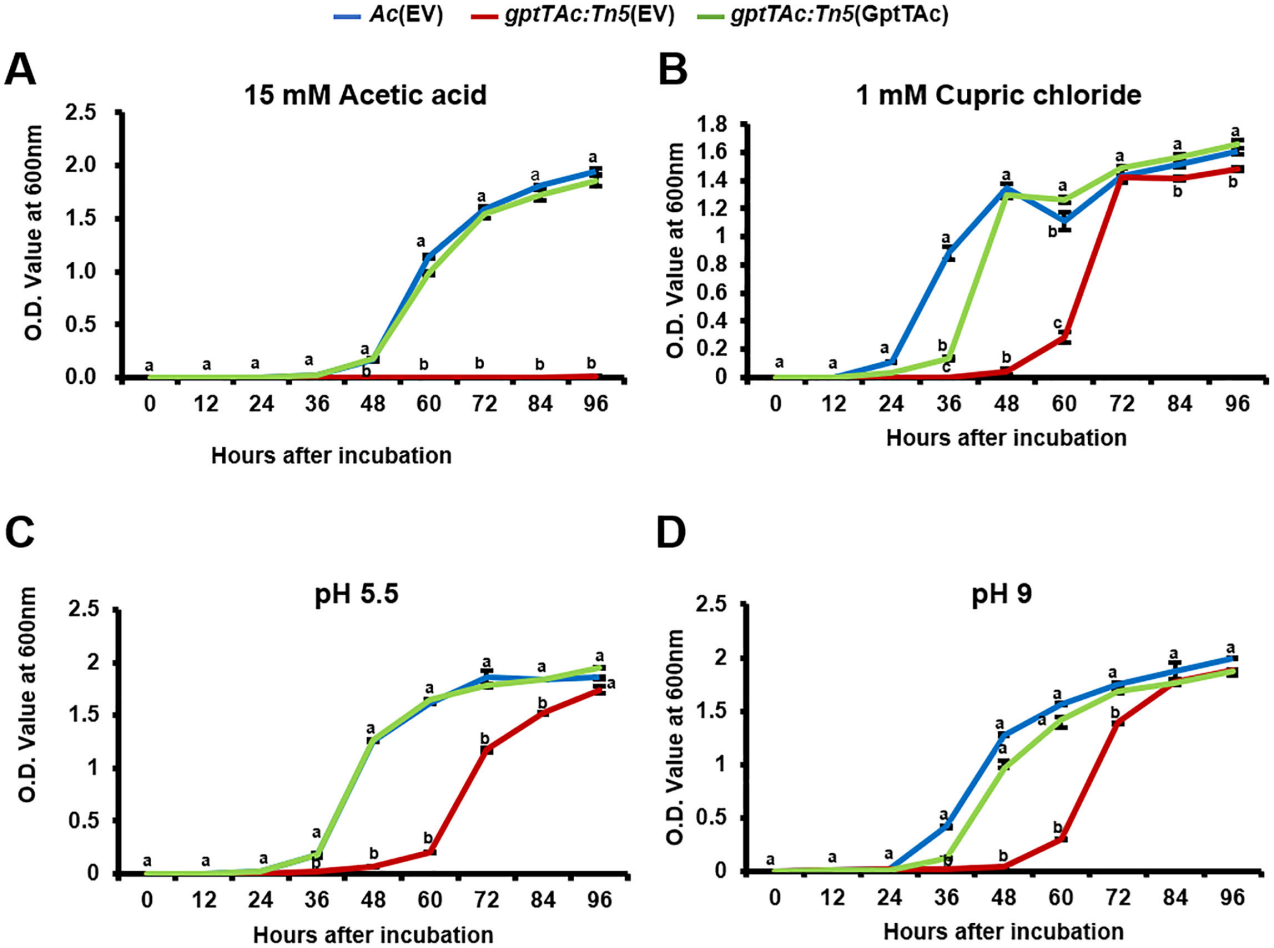
Growth assays of *Ac* strains exposed to acetic acid, cupric chloride, and acidic and basic conditions. *Ac*(EV), *gptTAc: Tn5*(EV), and *gptTAc: Tn5(*GptTAc) were incubated in TSB **(A)** with 15 mM acetic acid, **(B)** with 1 mM cupric chloride, and at pH **(C)** 5.5 and **(D)** 9. Bacterial growth was assessed by measuring the OD value at 600 nm for 94 h Alphabets above error bars (standard deviations) indicate statistical significance determined by ANOVA (p < 0.05) with Tukey’s HSD^ab^ test. At least three independent experiments were conducted, and all showed similar patterns.

The comparative proteomic analysis and the growth assays using various antibiotics revealed that GptTAc is essential for maintaining bacterial membrane integrity. Consequently, stress responses exposed to physical and chemical conditions were also investigated. Firstly, the growth patterns in the presence of acetic acid were analyzed (**Figure 7A**). Strikingly, the mutant strain failed to grow in the presence of 15 mM acetic acid. In contrast, both *Ac*(EV) and the complemented strain *gptTAc: Tn5*(GptTAc) grew after 36 h of incubation. Next, the impact of copper on bacterial growth was assessed using cupric chloride (**Figure 7B**). The wild-type strain exhibited an increase in population after 12 h of incubation, reaching the stationary phase by 48 h. Interestingly, *gptTAc: Tn5*(EV) required an extended incubation period (48 h) to enter the exponential phase and ultimately reached a population level comparable to the wild-type strain after 72 hours in the presence of 1 mM cupric chloride. The growth pattern of the complemented strain was restored to that of the wild-type in the presence of 1 mM cupric chloride. Finally, the effects of different pH levels, including acidic and basic conditions on the growth of *Ac* strains were examined. The growth patterns of the three strains, *Ac*(EV), *gptTAc: Tn5*(EV), and *gptTAc: Tn5*(GptTAc), were similar when incubated at pH 6, pH 8, and in TSB control (neutral) conditions (**Supplementary Figures 2**, **4**). Notably, at pH 5.5 and 9, the mutant strain showed significantly impaired growth compared to the wild-type and complemented strains (**Figures 7C, D**). However, none of the tested strains were able to grow at pH 5 or 10 (**Supplementary Figure 4**).

## Discussion

The deletion or inhibition of GptT has been found to significantly affect virulence in animal pathogenic bacteria (Qu et al., 2007; Alphey et al., 2013; Qu et al., 2021; Xiao et al., 2021). For example, the GptT mutant of *Mycobacterium tuberculosis* exhibited attenuated virulence during macrophage infection (Qu et al., 2021). Similarly, the virulence of *gptTAc: Tn5*(EV) lacking functional GptTAc was reduced in both germinated-seed inoculation and leaf infiltration assays (**Figure 1**), indicating that GptTAc is crucial for virulence in the plant pathogenic bacterium. In addition, GptT catalyzes the conversion of glucose-1-phosphate and dTTP to dTDP-glucose, which is the first step in the production of dTDP-L-rhamnose for the synthesis of LPS, a major virulence factor in gram-negative bacteria (Bertani and Ruiz, 2018; Brown et al., 2018). Besides, it is also reported that GptT is involved in the production of LPS and EPS (Zuccotti et al., 2001; Zivkovic et al., 2015). Interestingly, the pattern of LPS in *gptTAc: Tn5*(EV) was similar to that in the wild-type strain, indicating that GptTAc is not associated with the formation of basal structures of LPS (**Figure 3A**) Moreover, GptT is indispensable for mycobacterial growth (Qu et al., 2007). However, the growth of *gptTAc: Tn5*(EV) did not differ from that of the wild-type strain in both rich and minimal media (**Supplementary Figure 2**), indicating that GptTAc is not associated with bacterial multiplication and reproduction. Therefore, it can be postulated that the functions and related mechanisms of GptTAc differ from those of the previously characterized GptTs in animal pathogenic bacteria.

GptTs in other pathogenic bacteria, including *M. tuberculosis*, contribute to bacterial cell wall integrity (Brown et al., 2018; Qu et al., 2021). The comparative proteomic analysis performed in this study also suggested that GptTAc is associated with the functions of the cell wall and membrane in *Ac*. Moreover, the growth of *gptTAc: Tn5*(EV) was attenuated or reduced under diverse stress conditions. These results indicate that GptTAc is crucial for the integrity and stability of the cell wall/membrane in *Ac* because the bacterial cell/membrane serve as the first barrier to protect bacteria from external stress factors (Murinova and Dercova, 2014; Strahl and Errington, 2017). In the comparative proteomic analysis, a discernible difference was noted in the abundance of membrane-associated proteins (group M), including type II secretion system F family proteins, ABC transporter substrate-binding proteins, and ABC transporter ATP-binding proteins. These findings indicate the perturbation of essential elements, such as the ABC transporter system and ExbD, which are vital for diverse biological processes, including nutrient absorption, ion transport, and drug detoxification (Davidson and Chen, 2004; Braun et al., 2023). Consequently, the instability of the membrane and the altered abundance of membrane-associated proteins may affect the tolerance to external stresses in the mutant. This reinforces the hypothesis that issues pertaining to cell membrane integrity hinder bacterial adaptation to stress.

In addition to group M proteins, diverse proteins belonging to group N (motility), including PilT, pilY, PilB, PilO, and flagellin were detected in the comparative proteomic analysis, suggesting that GptTAc is related to motility in *Ac*. Because *Ac* strain KACC17005 used in this study belongs to group II, which only exhibits pilus-dependent motility but not flagellum-dependent motility *in vitro* (Bahar et al., 2010; Park et al., 2017), we investigated pilus-dependent motility. Indeed, the mutant exhibited reduced twitching motility (**Figures 4A, B**). Moreover, autoaggregation was enhanced in the mutant (**Figures 4C, D**). Aggregation is one of key determinants of biofilm formation, adhesion, and motility (Ruhal and Kataria, 2021; Feitosa-Junior et al., 2022). In agreement with our findings, a previous study reported that PilT is associated with twitching motility and autoaggregation (Bahar et al., 2009). It is well-established that bacterial motility plays a crucial role in biofilm formation (Ruhal and Kataria, 2021). Furthermore, autoaggregation is associated with biofilm formation (Feitosa-Junior et al., 2022). As expected, biofilm formation was enhanced in the mutant (**Figures 3F, G**). It can be speculated that abnormal autoaggregation in the mutant enhances biofilm formation.

In contrast to biofilm formation, EPS production was reduced in the mutant. While EPS is a crucial component of biofilm formation, EPS production and biofilm formation do not always show a direct correlation. For instance, Dertli et al. demonstrated that in *Lactobacillus johnsonii* the *epsE*-deficient mutant exhibited reduced production of EPS, but enhanced biofilm formation and autoaggregation (Dertli et al., 2015). Based on their findings and our observations, it can be postulated that reduced EPS may mask surface properties related to cell-cell interaction, leading to increased biofilm formation and autoaggregation. However, the precise mechanisms underlying the relationship between the reduction of EPS and the enhancement of biofilm formation remain unclear. Besides, motility and biofilm formation are often inversely regulated in bacteria (Guttenplan and Kearns, 2013). In other words, there appears to be a “motility-to-biofilm transition,” wherein the inhibition of motility promotes biofilm formation. Our results also revealed similar patterns (**Figures 3E, F**, **4A, B**), suggesting that reduced motility could enhance biofilm formation. Thus, reduced motility and abnormal biofilm formation may contribute to virulence in the mutant.

As mentioned, GptTs that are related to the biosynthesis of L-rhamnose in animal pathogenic bacteria serve as a vital component of the bacterial cell wall and membrane and form a crucial link between peptidoglycan and arabinogalactan layers, maintaining cell wall integrity (Brown et al., 2018; Qu et al., 2021). Lysozyme hydrolyzes the crucial beta-1,4-glycosidic bond required for the linkage between NAG and NAM, fundamental components of the peptidoglycan backbone (Wang et al., 2012). Consequently, the increased susceptibility of *gptTAc: Tn5*(EV) appears to stem from alterations in the outer membrane integrity resulting from cell membrane changes (**Figure 6A**). Penicillin is a β-lactam antibiotic that inhibits peptidoglycan synthesis and primarily targets gram-positive bacteria (Ligon, 2004). The wild-type strain was resistant to penicillin, while the mutant was highly sensitive to it (**Figure 6B**). Besides, vancomycin is a glycopeptide antibiotic that inhibits cell wall synthesis by disrupting the peptidoglycan structure in gram-positive bacteria; however, its efficacy in gram-negative bacteria is limited because of the challenge posed by their double membranes (Goldman et al., 2000; Antonoplis et al., 2019; Acharya et al., 2022). The sensitivity to vancomycin was significantly greater in *gptTAc: Tn5*(EV) than in the wild-type and complemented strains (**Figure 6E**). It can be speculated that GptTAc influences the integrity of membranes or peptidoglycan layers, leading to an abnormal phenotype in the mutant. Polymyxin B induces cell structure instability via electrostatic interactions with the lipid A component of LPS in gram-negative bacteria, eventually causing bacterial cell death (Poirel et al., 2017). As expected, the growth patterns of *Ac* strains in the presence of polymyxin B were similar to those in the presence of lysozyme, penicillin, and vancomycin. Consequently, the lack of GptTAc in the mutant likely weakens the functions of the bacterial cell wall/membrane, thereby increasing the sensitivity to diverse antibiotics targeting the bacterial membrane.

In addition to antibiotics, bacterial membranes are exposed to various physical and chemical stressors. SDS and EDTA are well-characterized membrane-disrupting agents that primarily target lipid components (Alakomi et al., 2006; Liu et al., 2023a). Our results showed that *gptTAc: Tn5*(EV) exhibited markedly increased sensitivity to both SDS and EDTA compared to the wild-type strain (**Figure 5**), indicating compromised membrane integrity. Similarly, the mutant completely lost tolerance to acetic acid and displayed significantly delayed growth in the presence of cupric chloride (**Figures 7A, B**). Acetic acid is known to disrupt membrane integrity (Ji et al., 2023), while copper, a widely used antimicrobial, induces rapid membrane damage (Mathews et al., 2013; Salah et al., 2021). Bacteria typically counter copper toxicity through outer membrane proteins that mediate copper ion binding, transport, and homeostasis (Bhamidimarri et al., 2021). Thus, the impaired growth observed in the mutant likely results from defective membrane function due to the loss of functional GptTAc. Furthermore, *gptTAc: Tn5*(EV) exhibited growth retardation under both acidic and alkaline conditions (**Figures 7C, D**), consistent with the role of membrane-associated pumps and transporters in pH regulation (Krulwich et al., 2011; Teelucksingh et al., 2020). Collectively, these findings implicate GptTAc as a critical factor in the maintenance of membrane integrity and functionality. Nonetheless, the precise molecular mechanisms underlying GptTAc function remain to be elucidated. Further investigations at the molecular and biochemical levels have to be conducted to elucidate its role in membrane-associated processes.

In this study, GptTAc, which serves as a central component in the dTDP-L-rhamnose biosynthesis pathway, was identified as an indispensable factor for the pathogenicity of *Ac*. Moreover, the functions of GptTAc and related mechanisms were identified by performing a comparative proteomic analysis. The results suggested that GptTAc is associated with diverse biological mechanisms, including bacterial motility and the cell wall and membrane. Finally, the functions of GptTAc, which is required for the integrity and stability of the bacterial cell wall/membrane, were assessed using diverse phenotypic assays. GptTAc influences LPS and EPS production, biofilm formation, twitching motility, autoaggregation, external stress response, and antibiotic resistance, which may contribute to virulence in the bacterium (**Supplementary Figure 5**). These findings provide new insights into the virulence mechanisms of *Ac*, potentially paving the way for novel strategies for controlling BFB. GptT has been thought to be a target for drug development against human pathogenic bacteria (Alphey et al., 2013; Brown et al., 2018). Accordingly, GptTAc may also serve as a promising target for the discovery of novel compounds capable of controlling BFB by suppressing bacterial virulence, through the screening of small molecule and natural product libraries.

## Data Availability

The datasets presented in this study can be found in online repositories. The names of the repository/repositories and accession number(s) can be found in the article/**Supplementary Material**.

## Supplementary material

Supplementary Figure 1 Amino acid sequence comparison and 3D structure analysis of GptTAc. **(A)** Comparison of the deduced amino acid sequences of glucose-1-phosphate thymidylyltransferase in *Acidovorax citrulli* KACC17005 (GptTAc, ATG96716), *Salmonella enterica* (EAO9629959), and *Escherichia coli* (MRF41702) using the Clustal Omega program. In the sequence alignment, “*” represents identical residues, “:” denotes conserved substitutions, and “.” indicates semiconserved substitutions. **(B)** Predicted 3D protein structures of GptTAc, EAO9629959, and MRF41702. PFB files obtained from the I-TASSER program were visualized using PyMOL.

Supplementary Figure 2 Growth curves of *Ac* strains in rich and minimal media. Growth curves of *Ac*(EV), *gptTAc: Tn5*(EV), and *gptTAc: Tn5(*GptTAc) were determined in **(A)** TSB for 96 h and **(B)** M9 for 8 days using a spectrophotometer at 600 nm. Error bars represent standard deviations. Alphabets above error bars (standard deviations) indicate statistical significance determined by ANOVA (p < 0.05) with Tukey’s HSD^ab^ test. At least three independent experiments were conducted, showing similar patterns.

Supplementary Figure 3 Autoaggregation in *Ac*(EV), *gptTAc: Tn5*(EV), and *gptTAc: Tn5(*GptTAc). Photographs showing aggregated cells at the bottom of 14 mL test tubes at 60 h after incubation.

Supplementary Figure 4 Growth curves of *Ac* strains under various pH conditions. *Ac*(EV), *gptTAc: Tn5*(EV), and *gptTAc: Tn5(*GptTAc) were incubated in TSB at pH **(A)** 5, **(B)** 6, **(C)** 8, and **(D)** 10. Bacterial growth was examined by measuring the OD value at 600 nm for 94 h. Alphabets above error bars (standard deviations) indicate statistical significance determined by ANOVA (p < 0.05) with Tukey’s HSD^ab^ test. At least three independent experiments were conducted, showing similar patterns.

Supplementary Figure 5 Schematic overview of the proposed functions and associated mechanisms of GptTAc in *Acidovorax citrulli*. Solid arrows indicate biological processes or phenotypes positively regulated by GptTAc, whereas dashed arrows represent processes that are negatively regulated.
