# Supplementary figures and images for: A putative glucose-1-phosphate thymidylyltransferase is required for virulence, membrane-associated mechanisms, and tolerance to external stresses in Acidovorax citrulli
Source: Front Plant Sci. 2025 May 21;16:1556578. doi: 10.3389/fpls.2025.1556578 (PMC12133956; doi:10.3389/fpls.2025.1556578)

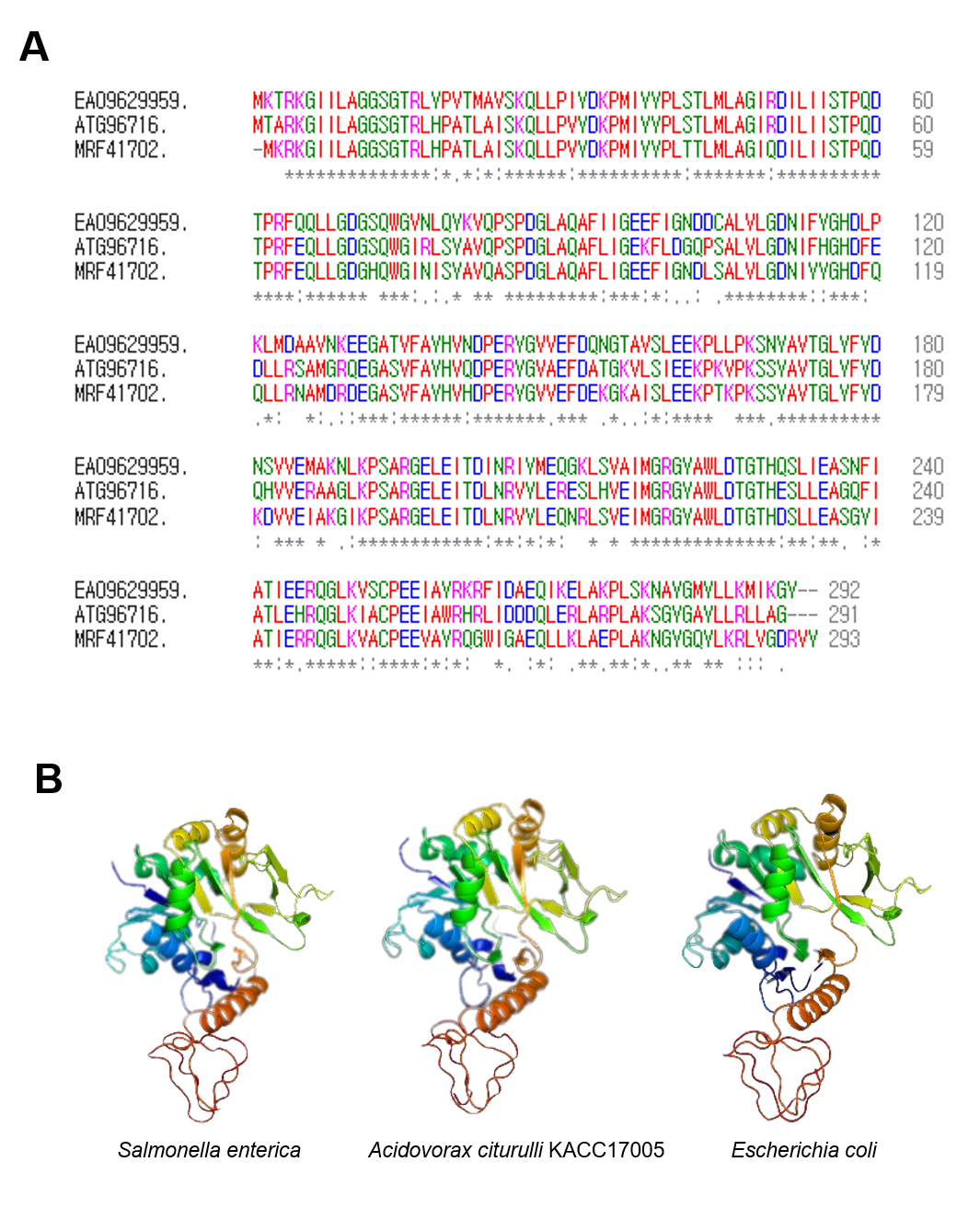

Supplement: Supplementary Figure 1 — Amino acid sequence comparison and 3D structure analysis of GptTAc. (A) Comparison of the deduced amino acid sequences of glucose-1-phosphate thymidylyltransferase in Acidovorax citrulli KACC17005 (GptTAc, ATG96716), Salmonella enterica (EAO9629959), and Escherichia coli (MRF41702) using the Clustal Omega program. In the sequence alignment, “*” represents identical residues, “:” denotes conserved substitutions, and “.” indicates semiconserved substitutions. (B) Predicted 3D protein structures of GptTAc, EAO9629959, and MRF41702. PFB files obtained from the I-TASSER program were visualized using PyMOL. [file Image1.tif]

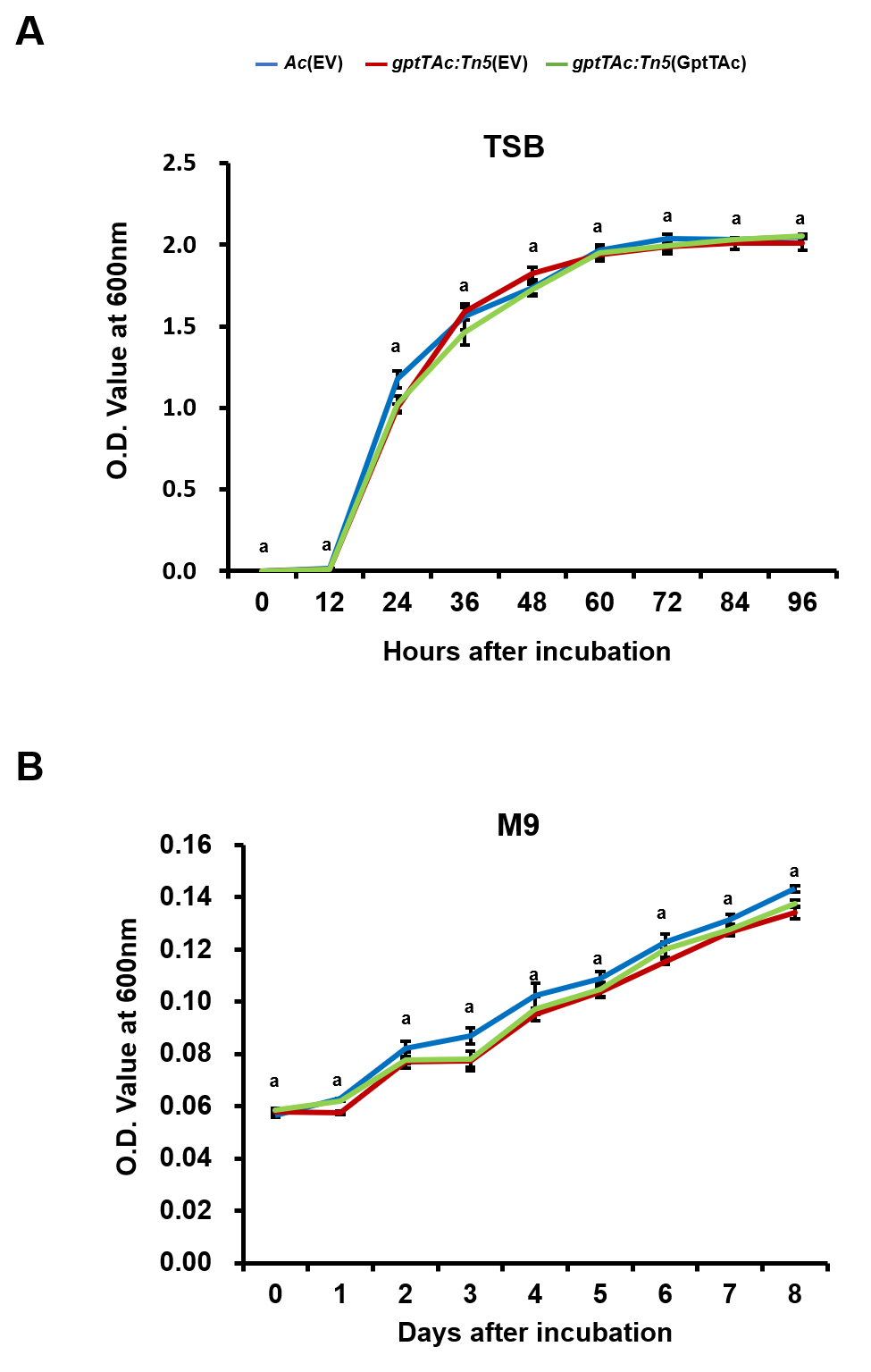

Supplement: Supplementary Figure 2 — Growth curves of Ac strains in rich and minimal media. Growth curves of Ac(EV), gptTAc: Tn5(EV), and gptTAc: Tn5(GptTAc) were determined in (A) TSB for 96 h and (B) M9 for 8 days using a spectrophotometer at 600 nm. Error bars represent standard deviations. Alphabets above error bars (standard deviations) indicate statistical significance determined by ANOVA (p < 0.05) with Tukey’s HSDab test. At least three independent experiments were conducted, showing similar patterns. [file Image2.tif]

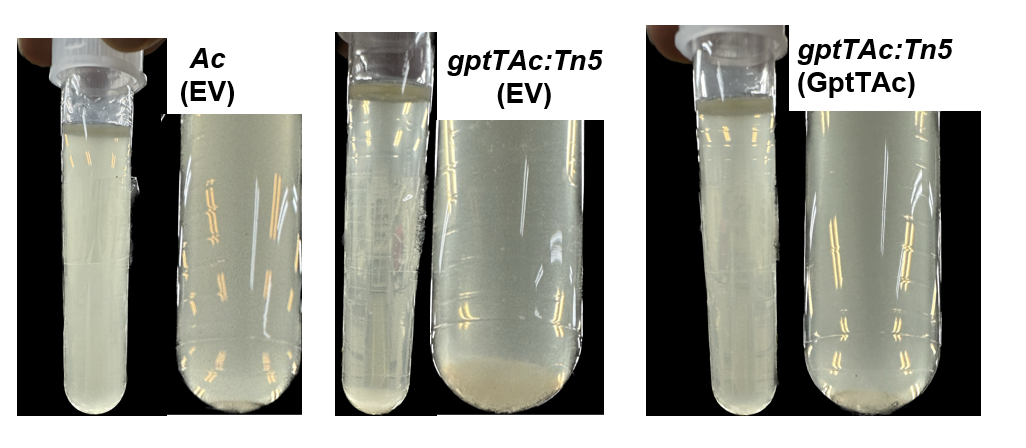

Supplement: Supplementary Figure 3 — Autoaggregation in Ac(EV), gptTAc: Tn5(EV), and gptTAc: Tn5(GptTAc). Photographs showing aggregated cells at the bottom of 14 mL test tubes at 60 h after incubation. [file Image3.tif]

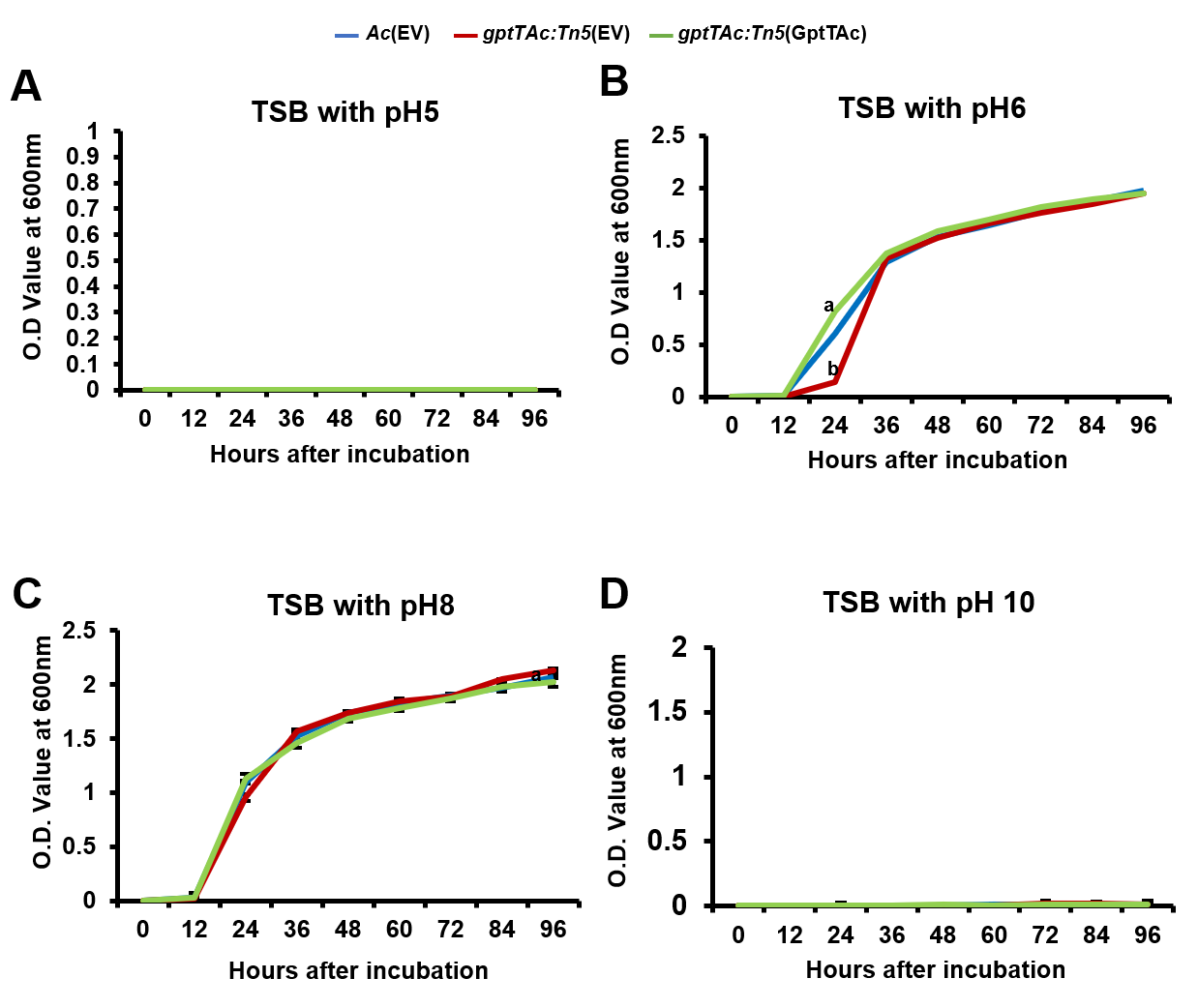

Supplement: Supplementary Figure 4 — Growth curves of Ac strains under various pH conditions. Ac(EV), gptTAc: Tn5(EV), and gptTAc: Tn5(GptTAc) were incubated in TSB at pH (A) 5, (B) 6, (C) 8, and (D) 10. Bacterial growth was examined by measuring the OD value at 600 nm for 94 h. Alphabets above error bars (standard deviations) indicate statistical significance determined by ANOVA (p < 0.05) with Tukey’s HSDab test. At least three independent experiments were conducted, showing similar patterns. [file Image4.tif]

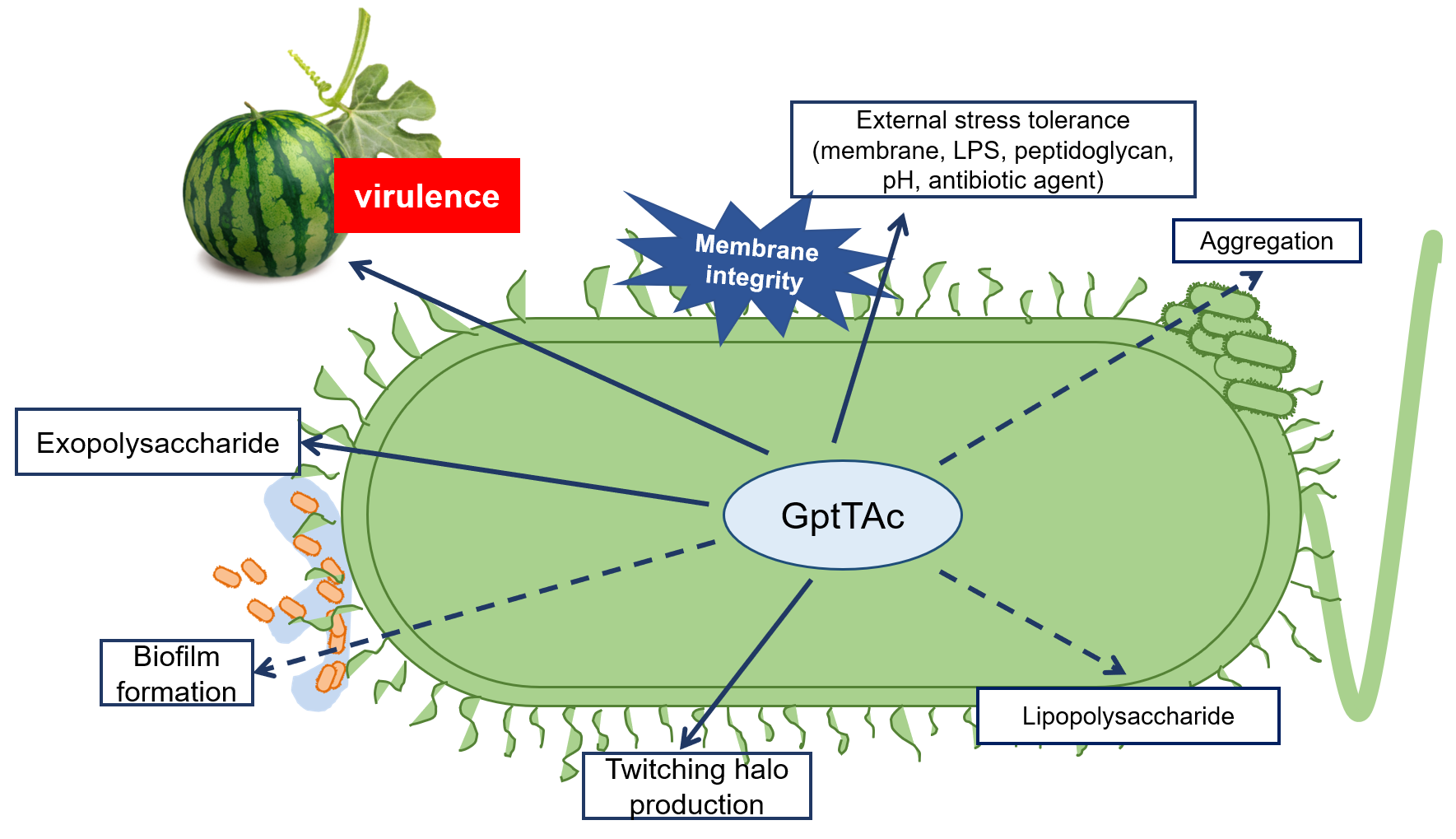

Supplement: Supplementary Figure 5 — Schematic overview of the proposed functions and associated mechanisms of GptTAc in Acidovorax citrulli. Solid arrows indicate biological processes or phenotypes positively regulated by GptTAc, whereas dashed arrows represent processes that are negatively regulated. [file Image5.tif]
