# Supplementary material for: A putative glucose-1-phosphate thymidylyltransferase is required for virulence, membrane-associated mechanisms, and tolerance to external stresses in Acidovorax citrulli
Source: Front Plant Sci. 2025 May 21;16:1556578. doi: 10.3389/fpls.2025.1556578 (PMC12133956; doi:10.3389/fpls.2025.1556578)
Supplement: Supplementary file 6 [file Table1.docx]

| **Supplementary Table 1. Bacterial strains and plasmids used in this study** | | |
| --- | --- | --- |
| Strains or plasmids | Characteristic(s) | Source or reference |
| *Escherichia coli* |  |  |
| EC100D | For identifying Tn5-insertional sites | Epicentre |
| DH5α | For cloning | Promega |
|  |  |  |
| *Acidovorax citrulli* |  |  |
| KACC17005 | Wild-type, Rif^r^ | (Park et al., 2017) |
| *Ac*(EV) | Wild-type carrying the pBBR1-MCS5, Rif^r^, Gm^r^ | This study |
| *gptTAc:Tn5* | Knockout mutant, Tn5-inserted in 394 bp from the start codon in *gptTAc* gene, Rif^r^, Kan^r^ | This study |
| *gptTAc:Tn5*(EV) | *gptTAc:Tn5* carrying the pBBR1-MCS5, Rif^r^, Kan^r^, Gm^r^ | This study |
| *gptTAc:Tn5*(GptTAc) | Complemented strain, *gptTAc:Tn5* carrying the MCS5-GptTAc, Rif^r^, Kan^r^, Gm^r^ | This study |
|  |  |  |
| Plasmids |  |  |
| pGem-T easy | TA cloning vector, Amp^r^ | Promega |
| pGem-*gptTAc* | pGem-T easy vector ligating 876 bp of *gptTAc* gene, Amp^r^ | This study |
| pBBR1-MCS5 | Broad-host-range vector, *LacZ* promoter, Gm^r^ | (Kovach *et al.*, 1995) |
| pMCS5-GptTAc | pBBR1-MCS5 carrying the *gptTAc* gene from pGem-*gptTAc* plasmid, Gm^r^ | This study |
| Rif^r^, Kan^r^, Gm^r^ and Amp^r^ represent resistance to Rifampicin, Kanamycin, Gentamycin, and Ampicillin, respectively. | | |
