# Supplementary material for: A putative glucose-1-phosphate thymidylyltransferase is required for virulence, membrane-associated mechanisms, and tolerance to external stresses in Acidovorax citrulli
Source: Front Plant Sci. 2025 May 21;16:1556578. doi: 10.3389/fpls.2025.1556578 (PMC12133956; doi:10.3389/fpls.2025.1556578)
Supplement: Supplementary file 7 [file Table2.docx]

**Supplementary Table 2. Proteins and peptide spectrum matches (PSM) between *Ac* and *gptTAc:Tn5***

| Strain | 1st | | 2nd | | 3rd | | shared proteins in 3 biological replicates |
| --- | --- | --- | --- | --- | --- | --- | --- |
|  | **Protein** | **PSM** | **Protein** | **PSM** | **Protein** | **PSM** |  |
| *Ac* | 723 | 28,289 | 723 | 28,287 | 723 | 28,287 | 687 |
| *gptTAc:Tn5* | 707 | 29,202 | 715 | 29,218 | 710 | 29,214 | 691 |
